# Rehabilitation of executive function in chronic paediatric brain injury: a randomized controlled trial

**DOI:** 10.1186/s12916-021-02129-8

**Published:** 2021-11-02

**Authors:** Anne E. Brandt, Torun G. Finnanger, Ruth E. Hypher, Torstein B. Rø, Eva Skovlund, Stein Andersson, Kari Risnes, Jan Stubberud

**Affiliations:** 1 Children’s Clinic, St. Olavs Hospital, Trondheim University Hospital, Postbox 3250 Torgarden, NO-7006 Trondheim, Norway; 2 Department of Clinical and Molecular Medicine, Norwegian University of Science and Technology, Trondheim, Norway; 3 Department of Clinical Neurosciences for Children, Oslo University Hospital, Oslo, Norway; 4 Department of Public Health and Nursing, Norwegian University of Science and Technology, NTNU, Trondheim, Norway; 5 Department of Psychology, University of Oslo, Oslo, Norway; 6 Department of Research, Lovisenberg Diaconal Hospital, Oslo, Norway

**Keywords:** Executive function, Cognition, Paediatric acquired brain injury, Goal management training, Cognitive rehabilitation

## Abstract

**Background:**

Impaired executive functions (EFs, i.e., purposeful, goal-directed behaviour) cause significant disability after paediatric acquired brain injury (pABI) warranting efficient interventions. Goal Management Training (GMT) is a metacognitive protocol proven effective for executive dysfunction in adults. This pre-registered, blinded, parallel-randomized controlled trial evaluated efficacy of a paediatric adaptation (pGMT) compared to a psychoeducative control (paediatric Brain Health Workshop, pBHW) to improve EF.

**Methods:**

Children aged 10 to 17 years with pABI (e.g., traumatic brain injury, brain tumour), ≥ 1 year post-onset or ended treatment, with parent-reported EF complaints were eligible. Participants were randomized (computer-algorithm) to either group-based pGMT (*n* = 38) or pBHW (*n* = 38). The active control was tailored to keep non-specific factors constant. Thus, both treatments comprised of 7 sessions at hospitals over 3 consecutive weeks, followed by 4 weeks of telephone counselling of participants, parents, and teachers. Parent-reported daily life EF, assessed by the questionnaire Behavior Rating Inventory of Executive Function (BRIEF; Behavioral Regulation Index (BRI) and Metacognition Index (MI)), were co-primary outcomes 6 months post-intervention. Secondary outcomes included neuropsychological tests and a complex naturalistic task (Children’s Cooking Task).

**Results:**

Seventy-three participants (96%) completed allocated interventions and 71 (93%) attended the 6-month follow-up. The results demonstrated no significant difference in effectiveness for the two interventions on parent-reported EF: For BRIEF_BRI_, mean (SD) raw score for pGMT was 42.7 (8.8) and 38.3 (9.3) for pBHW. Estimated difference was − 2.3 (95% CI − 5.1 to 0.6). For BRIEF_MI_, the corresponding results were 80.9 (20.4) for GMT and 75.5 (19.3) for pBHW. Estimated difference was − 1.4 (95% CI −8.5 to 5.8). In performance-based tests, pGMT was associated with improved inhibition and executive attention, while pBHW was associated with fewer errors in the naturalistic task.

**Conclusions:**

In pABI, metacognitive training (pGMT) did not demonstrate additional effectiveness on parent-reported daily life EF at 6-month follow-up, when compared to a psychoeducative control. Both interventions were well-tolerated and demonstrated distinct improvements at different EF assessment levels. To conclude on pGMT efficacy, larger studies are needed, including further investigation of appropriate assessment levels and possible differences in effect related to treatment duration, developmental factors, and injury characteristics.

**Trial registration:**

ClinicalTrials.gov, NCT0321534211, 11 July 2017

**Supplementary Information:**

The online version contains supplementary material available at 10.1186/s12916-021-02129-8.

## Background

Paediatric acquired brain injuries (pABIs), either traumatic brain injury (TBI) or non-traumatic (e.g., tumour, cerebrovascular accident, infection) are leading causes of childhood morbidity, mortality [1], and acquired disability [2]. As pABI occurs during crucial brain development, the consequences go beyond the immediate brain injury, affecting social competence, behavioural functioning, and cognition [3–5]. Indeed, executive dysfunction represents one of the most common and disturbing cognitive symptoms after pABI. Executive function (EF) refers to cognitive processes responsible for purposeful, goal-directed behaviour [6], operationalized [7, 8] in terms of three interrelated core processes: (a) *updating* (adding relevant and omitting non-relevant information from working memory), (b) *shifting* (switching between task sets), and (c) *inhibition* (suppressing or resisting pre-potent responses) [7–10]. Executive dysfunction has a substantial global negative impact on everyday life [11–14]. Despite this, there is no consensus regarding cognitive rehabilitation of EF following pABI [15–17].

There is solid empirical support for group-based cognitive interventions for adult ABI [18], with Goal Management Training (GMT) as one of the best validated protocols [19]. The theoretical foundation of GMT holds that the sustained attention system upholds higher-order goals in mind while inhibiting automatic processes [20, 21]. GMT addresses both core (e.g., inhibition and attention) and metacognitive processes (e.g., problem solving), a duality considered especially efficient [22]. Indeed, the effectiveness of GMT has been demonstrated across adult aetiologies, with reports of improved sustained and executive attention [23], assumed essential for daily life EF [24, 25] and for global outcomes such as education and independence [12, 26, 27]. Of note, paediatric GMT (pGMT) have been piloted [28] and found to be both feasible and acceptable [29].

The present study addressed previous methodological shortcomings by employing a robust RCT design including blinded assessments, long-term follow-up, active involvement of parents, and counselling of teachers [17, 30–32] as it is imperative to teach and support EF skills in the context of everyday activities and in close cooperation with the adults in the child’s life [30, 33]. Most pABI research focuses on single aetiologies [34]. However, as GMT has shown trans-diagnostic effects, the findings suggest that the intervention may be effective across paediatric aetiologies. Due to its multifaceted nature, the assessment of EF is challenging [7, 35–38]. Hence, comparing research employing conventional performance-based tests to studies employing rating scales involves uncertainty, as to whether they index the same underlying EF constructs [39]. Moreover, as GMT targets several EF aspects pertaining to both core processes (bottom-up) and metacognition (top-down), it addresses all levels of functional ability [38]. Thus, assessment should take into account the multifaceted nature of EF, addressing different aspects of EF potentially affected by treatment instead of only addressing one [35, 40]. Consequently, we used the International Classification of Functioning, Disability and Health (ICF) [41] as the basis to choose the outcome instruments: questionnaire (activity), performance-based neuropsychological tests (impairment) [42], and the novelty of adding a practical task (participation) [43].

The aim of the present study was to examine the efficacy of the metacognitive group-based pGMT, proven efficient in adults, for children in the chronic phase of pABI and with reported daily life executive difficulties. As a comparator, we chose a previously used group-based active control intervention (psychoeducation; paediatric Brain Health Workshop, pBHW) that was specifically tailored to keep non-specific factors constant (e.g., same therapists, corresponding structure, and duration of training and involvement of parents and teachers). Based on adult studies, we expected greater improvement in executive dysfunction in the pGMT group compared to the pBHW group. The two sub-indexes of the Behavior Rating Inventory of Executive Function (BRIEF, parent report) were co-primary outcomes. Secondary outcome measures were neuropsychological tests assessing core EF processes [7, 44], as well as a practical complex naturalistic task [45].

## Methods

### Trial design

This is an evaluator-blinded, parallel-RCT, with a previously published trial protocol [46], set at two paediatric hospitals in Norway, St. Olavs hospital, Trondheim University Hospital and Oslo University Hospital, Rikshospitalet. The sample size was initially calculated using the Global Executive Composite (GEC) from the BRIEF (parent report) [47], assuming an effect size of .70, significance level of .05, 80% power, and a *t*-test for difference in means, yielding samples of 32 individuals per group. Later, however, the Behavioral Regulation (BRI) and Metacognition (MI) indexes from BRIEF (that form the GEC), were considered to be more appropriate as co-primary outcomes. For practical and logistical reasons, it was not feasible to recalculate and increase the sample size of the study. Therefore, this study should not be considered as confirmatory as it is likely to be underpowered to find differences between the two groups, if they exist.

Participants were randomized to either pGMT or pBHW in a 1:1 ratio, by blocks of 4, and stratified by hospital site (Trondheim vs Oslo) and age at intervention (10–13 years vs 14–17 years). A computer-based algorithm was set up for randomization by an independent allocator. The following blinding procedures were applied to reduce systematic bias: (i) Treatment allocation was not disclosed to participants and families, and they were encouraged not to discuss course content outside their group. (ii) “Brain training” was used consistently in both groups. (iii) Independent, trained, and blinded test technicians conducted assessments. (iv) Group therapists were blinded to all assessments. (v) Blinded data monitoring and statistical analysis were implemented. Finally, interventions were arranged in random order.

Written informed consent was provided from primary caregivers of participants (< 16 years) and from participants and their caregivers (> 16 years). Study procedures and monitoring according to Norwegian Clinical Studies Infrastructure Network procedures.

### Recruitment and eligibility

**Fig. 1:**
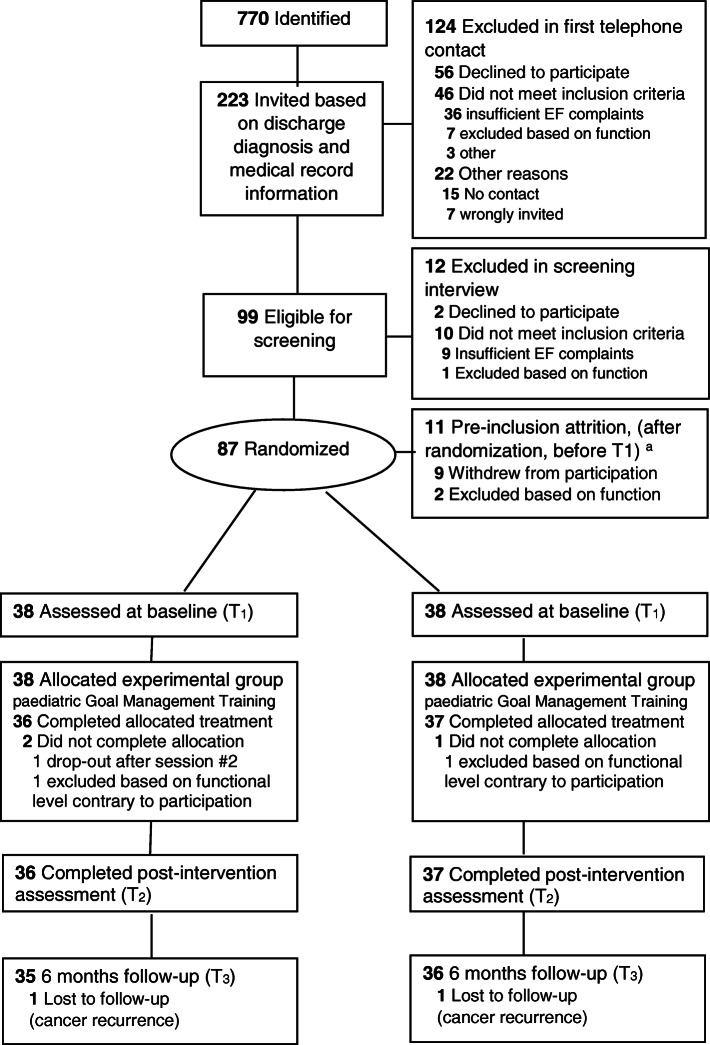
Consort flow diagram. ^a^Nine withdrew while waiting for start of assigned intervention group (worsening of illness, medication testing, intensifying physical rehabilitation), 2 were excluded post-randomization (before baseline) after identification of violations of eligibility criteria not previously communicated. Attrition distribution was evenly distributed between the two groups, 6 in pGMT and 5 in pBHW. See also Additional file 4, Consort checklist

Participants eligible for a written invitation were identified based on discharge diagnosis and medical records (Fig. 1) [46]. Further eligibility screening was done in an initial telephone contact and more thoroughly in a semi-structured screening interview [see Additional file 1] with primary caregivers (or participants surpassing 16 years). Successively, after written informed consent, eligible individuals were designated a study number and randomized.

Eligible children were aged 10 to 17 years, diagnosed with pABI (TBI, brain tumour, cerebrovascular accident, hypoxia/anoxia, or brain infection/inflammation), at intervention > 12 months post-insult or > 12 months stable brain tumour situation/ended cancer therapy, with reported executive function difficulties in daily life as described in the screening interview by parents (or participants). Exclusion criteria included pABI before the age of 2 years; cognitive (including memory), physical, or language impairments affecting the capacity to follow educational goals of peers and attend regular classroom teaching; pre-injury neurological disease; severe psychiatric disorder and/or on stimulant medication; recently detected brain tumour relapse; or not fluent in Norwegian. To our knowledge, none of the participants had prior to the study received specific cognitive rehabilitation. Recruitment lasted from November 2017 until May 2019 and was ended at achieved sample size.

### Interventions

The two group-based interventions compared in this study were the metacognitive pGMT and an active control (pBHW) involving the use of educational materials and lifestyle topics, similar to many other psychoeducational ABI rehabilitation programmes [48]. While the pBHW programme was not specifically aimed at remediating EF, the pBHW programme was tailored to keep non-specific factors constant (i.e., structure and intensity/duration of training, between session-assignments, involvement of parents and teachers, professional attention, group dynamics).

### Paediatric goal management training

**Fig. 2:**
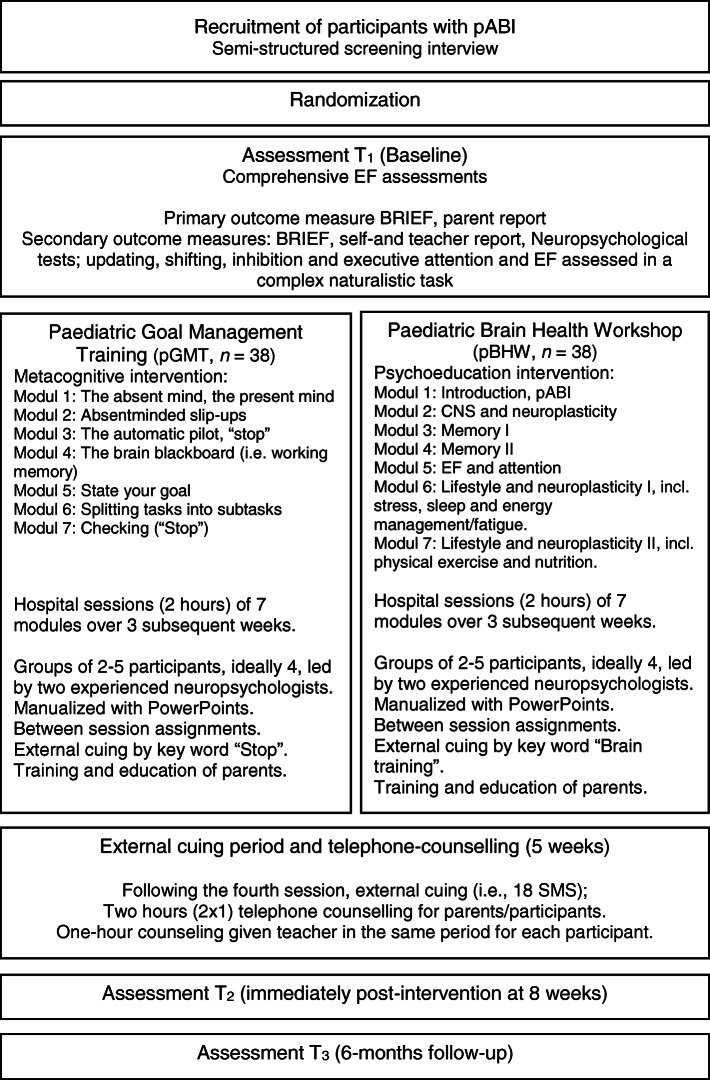
Outline of the study design. pABI, paediatric acquired brain injury; EF, executive function; BRIEF, Behavior Rating Inventory of Executive Function; pGMT, paediatric Goal Management Training; pBHW, paediatric Brain Health Workshop

The manualized GMT®, adult protocol has previously been translated into Norwegian [49, 50]. A preliminary paediatric protocol was developed and piloted by our research group [29] and then further refined based on the feedback for the present study. The pGMT promotes inhibitory control and metacognitive strategies and facilitation of goal-achievements, in addition to mindfulness training [51]. The protocol consists of 7 sessions, each of 2-h duration (Fig. 2). Original GMT materials were made more age-appropriate (e.g., examples and discussions more related to school and leisure activities, children portrayed in illustrations instead of adults, language simplified, more visual materials, and movie clips were included).

### Active control intervention

The original adult Brain Health Workshop (BHW) protocol is a manualized psychoeducational intervention developed to match GMT for non-specific factors [20, 24], and adapted into a paediatric version in a similar manner as described for pGMT. pBHW represents an active control intervention including educational materials addressing brain injury and (dys)function, plasticity, memory and learning, EF, fatigue, and lifestyle issues (e.g., stress, sleep, exercise and nutrition) [20, 24] (Fig. 2).

### Similarities of treatments for non-specific factors and treatment fidelity

The two group-based interventions were corresponding regarding structure, intensity, duration, therapist contact, and access to distributed materials. Treatment fidelity was ensured by two trained therapists at each site and standardization of materials to warrant consistent and reliable implementation. Accompanying all sessions, pre-prepared manualized PowerPoint slides were used to standardize content across groups and sites. Additionally, participant workbook inserts specific for each session were handed out at the start of each session. Both interventions included discussions of participants’ real-life experiences and attention deficits, in-session practice, between-sessions exercises, monitoring of activity, education of parents and teachers, and periodic alertness cueing following the fourth session (SMS; stating the key term “STOP” in pGMT and the more general “Brain training” in pBHW) to facilitate transfer to daily living [52, 53]. If a participant was prevented from attending a group session, an individual session of the missed module was offered before the following to ensure progress. Parents received a 1-h review after every paediatric session with group therapists allowing questions and exchange of experience. In the 4 weeks following sessions, telephone counselling was conducted to promote use of the interventions. All designated teachers received written information about the study and a 1-h telephone counselling post sessions that included a brief review and encouragement to implement intervention principles [30]. Last follow-up was conducted in November 2019.

### Baseline assessment

Medical characteristics at the time of insult were extracted from the medical records (Table 1). At start of intervention (*T*_1_), systematic assessments of baseline sociodemographic factors and medical history as well as a standardized physical examination was performed for all individuals by a study nurse and a paediatrician. Fatigue was measured with the Pediatric Quality of Life Inventory-Multidimensional Fatigue Scale (PedsQL MFS, parent report) [54]. Parents rated each item according to their child’s function the prior month (e.g., “Feels too tired to spend time with his/her friends”). Presented are reversed total score, linearly transformed to a 0–100 scale. A higher score indicates fewer fatigue symptoms. General intellectual ability was measured by full scale intelligence quotient (FSIQ) from Wechsler Intelligence Scale for Children-Fifth edition (WISC-V), with normalized IQ distribution (*M* = 100, SD *=* 15). Blinded baseline assessment was performed the day prior to initiation of the interventions as several participants had a long commute to the research site and reported fatigue, thus avoiding the burden of an additional travel just for the assessment.

**Table 1:** Participant demographics, injury characteristics and cognitive scores at baseline (*T*_1_)

	Interventions		Total (*n* = 76)
	pGMT (*n* = 38)	pBHW (*n* = 38)	
**Demographics**			
Sex, girls, No. (*%*)	21 (55)	22 (58)	43 (57)
Maternal educational level, No. (*%*) ^a^			
Primary school	2 (5)	2 (5)	4 (5)
High school	9 (24)	14 (37)	23 (30)
University/college	25 (66)	21 (55)	46 (61)
Intact family unit, No. (*%*)	24 (63)	26 (68)	50 (66)
Age at intervention, No. (*%*)			
10–13 years	20 (53)	20 (53)	40 (53)
14–17 years	18 (47)	18 (47)	36 (47)
Hospital site, No. (*%*)			
St. Olavs Hospital Trondheim	20 (53)	22 (58)	42 (55)
Oslo University Hospital	18 (47)	16 (42)	34 (45)
**Injury characteristics**			
Age at injury, median (*IQR*), years	8.4 (5.3–10.9)	8.5 (6.3–11.1)	8.5 (5.9–11)
Time since injury, median (*IQR*), years	4.8 (2.8–6.6)	5.5 (3.3–7.5)	5.3 (3.3–7.3)
Primary injury, No. (*%*)			
Brain tumour	15 (40)	14 (37)	29 (38)
Traumatic brain injury	7 (18)	11 (29)	18 (24)
Cerebrovascular accidents	10 (26)	7 (18)	17 (22)
Infection/Inflammation	3 (8)	4 (11)	7 (9)
Hypoxia/Anoxia	3 (8)	2 (5)	5 (7)
Admitted to intensive care unit, No. (*%*)	25 (66)	24 (65)	49 (65)
No. of days in intensive care unit, median (*IQR*)	4.0 (1.0–7.0)	2.0 (1.0–7.0)	2.0 (1.0–7.0)
Cerebral imaging, No. (*%*) ^b^	38 (100)	38 (100)	76 (100)
Confirmatory findings, No. (*%*) ^c^	36 (95)	31 (82)	67 (88)
Pathological neurological findings at baseline, No. (*%*)	20 (53)	13 (34)	33 (43)
Fatigue at baseline, reversed total score	54.7 (19.3)	55.9 (19.3)	55.3 (19.2)
**Cognitive scores** ^**d**^, mean (*SD*)			
Intellectual ability (FSIQ), age normed (*n* = 72)	92.5 (13.2)	92.4 (13.6)	92.5 (13.3)
BRIEF_BRI_ parent, *T*-score (*n* = 76)	57.2 (9.4)	55.4 (15.1)	56.3 (12.5)
BRIEF_MI_ parent, *T*-score (*n* = 75)	62 (9.6)	60.1 (11.3)	61.1 (10.4)
BRIEF_BRI_ self-report *T*-score (*n* = 75)	50.9 (11.7)	52.1 (12.1)	51.5 (11.8)
BRIEF_MI_ self-report, *T*-score (*n* = 75)	55.7 (12.7)	57.2 (12.7)	56.4 (12.6)
BRIEF_BRI_ teacher report, *T*-score (*n* = 72)	57.6 (15.9)	59.3 (18.0)	58.5 (16.9)
BRIEF_MI_ teacher report, *T*-score (*n* = 71)	61.5 (11.8)	61.5 (16.5)	61.5 (14.3)
Updating, scaled score (*n* = 73)	9.3 (2.9)	8.5 (2.8)	8.9 (2.8)
Shifting, scaled score (*n* = 76)	8.5 (3.9)	7.8 (3.5)	8.1 (3.7)
Inhibition, *T*-score (*n* = 76)	51.3 (8.6)	54 (7.7)	52.6 (8.3)
Executive attention, scaled score (*n* = 76)	7.4 (3.9)	8.0 (3.2)	7.7 (3.6)
Complex naturalistic task, total errors (*n* = 72)	27.4 (19.6)	29 (23.7)	28.3 (21.7)

*pGMT* paediatric Goal Management Training, *pBHW* paediatric Brain Health Workshop, *IQR* Interquartile range, *BRIEF* Behavior Rating Inventory of Executive Function, *BRI* Behavioral Regulation Index, *MI* Metacognition Index

^a ^Only 73 out of 76 mothers (96%) stated their level of education

^b^All participants had conducted magnetic resonance imaging (MRI) or computed tomography (CT) at some point. Of these, 4 individuals (11%) in the pGMT group and 7 (18%) in the pBHW group had only performed CT

^c ^Out of the 9 individuals without confirmatory imaging (normal), 6 had sustained TBI, 1 hypoxia, and 2 with brain infection/inflammation. Three out of 9 participants without confirmatory imaging had conducted a CT only and all of these had TBI. ^d^ Fatigue is presented as reversed total score, linearly transformed to a 0–100 scale. A higher score indicate fewer fatigue symptoms

^e ^Specifications cognitive measures

Intellectual ability: Full Scale Intelligence Quotient (FSIQ) from Wechsler Intelligence Scale for Children-Fifth edition (WISC-V) with normalized IQ distribution (*M* = 100, SD = 15). Only 72 out of 76 managed to complete the required number of tests in WISC-V to calculate FSIQ. BRIEF_BRI_ and BRIEF_MI_ (*T*-scores, normative: *M* = 50, SD = 10). Update: digit span, from WISC-V (scaled score, normative: *M* = 10, SD = 3); shift: Trail Making Test 4 from the Delis–Kaplan Executive Function System (D-KEFS) (total time, scaled score, normative: *M* = 10, SD = 3); inhibition: commissions from Conners’ Continuous Performance Test, 3ed (CPT-III) (*T*-scores, normative: *M* = 50,SD = 10); and executive attention: Color Word Interference Test 4 (CWIT4, D-KEFS) (total time, scaled score, normative: *M* = 10, SD = 3). Complex naturalistic task: Children’s Cooking Task (CCT) (raw score, total errors)

### Outcome measures

An accurate and valid evaluation of EF benefits from assessments at different levels to obtain clinically useful functional ability information [38, 55]. Since pGMT addresses all three ICF- levels [41], EF outcome measures pertaining the three levels were included by questionnaire (activity level, primary outcome measure), performance-based tests (impairment level, secondary outcome measure), and a complex naturalistic task (participation level, secondary outcome measure) [38, 43, 56]. Investigators blinded to the intervention performed assessments at pre-intervention (baseline, *T*_1_), post-intervention (*T*_2_), and at 6-month follow-up (*T*_3_).

The BRIEF parent report [47] at *T*_3_ was employed as the primary EF outcome measures using the two indexes, the BRI and the MI as co-primary endpoints (raw scores BRIEF_BRI_ and BRIEF_MI_). Since the BRIEF has been used as a primary outcome in adult GMT studies [49] results can potentially be compared. The BRIEF is an 86-item standardized questionnaire designed to capture parent perceptions of a child’s everyday EF. Each item’s frequency of occurrence is rated on a 3-point Likert scale from 1 (never) to 3 (often), higher scores indicate greater dysfunctions. The BRIEF has proven applicable to several clinical groups [57] and has demonstrated adequate internal consistency, inter-rater and test-retest reliability [47, 58] for parent report, in addition to self- and teacher reports (secondary outcomes).

Secondary outcome assessments of EF at the impairment level were conducted with standardized neuropsychological tests pertaining updating (digit span, total score, raw score (Wechsler Intelligence Scale for Children-Fifth edition, WISC-V)) [59]; shifting (Trail Making Test 4 (TMT 4); total time; raw scores from the Delis–Kaplan Executive Function System (D-KEFS) [60]; inhibition (Conners’ Continuous Performance Test, 3ed (CPT-III) Commissions, *T*-scores) [61]; and executive attention (Color Word Interference Test 4 (CWIT 4, D-KEFS), total time, raw scores) [60]. All tests are paper and pencil tests, except CPT-III, which is a computerized assessment of different aspects of attention. Higher test scores on CPT-III and digit span indicated better performance, whereas higher scores on TMT 4 and CWIT 4 indicated poorer performance.

Finally, at the participation level, we included a complex open-ended task, the Children’s Cooking Task (CCT), total errors, and raw scores [45, 62]. The task (cooking) required multitasking in a naturalistic setting (kitchen) with aspects of novelty and distractions and, hence, less external control and structure. The CCT has demonstrated sensitivity to executive dysfunction in pABI [63].

### Statistical analysis

Categorical data were summarized by counts and percentages. Continuous data were summarized by mean and standard deviation (SD) or median and interquartile range (IQR), presented for each group. Any baseline differences between the groups were viewed as incidental [64] due to the randomization, but noted. A statistical analysis plan (SAP) was outlined in advance and registered at ClinicalTrials.gov prior to un-blinding of the data. The primary analysis was carried out blind of allocation by a statistician. The within and between differences of the two co-primary endpoints constituting BRIEF (raw scores BRIEF_BRI_ and BRIEF_MI_) were estimated by linear mixed models [65] in a full analysis set (FAS). All randomized individuals with post-baseline outcome data (ICH E9) [66] were included, representing an intention-to-treat approach. The model included baseline raw scores as a covariate [67], and treatment group, time (post-treatment (*T*_2_) and at 6 months follow-up (*T*_3_)), interaction between time and treatment, and randomization strata (1) site (Trondheim or Oslo) and (2) age at intervention (10–13 or 14–17 years) as fixed factors. Time was included as categorical with unstructured covariance as the starting point. According to the SAP, the per-protocol population included participants who had missed a maximum of 2 out of the 7 group sessions. Since all participants who completed the allocated intervention (*n* = 73) underwent all 7 modules the main analysis (FAS) and per-protocol were identical. The two co-primary endpoints from BRIEF (_BRI_ and _MI_) were analysed using the Hochberg procedure to control for the type I error rate [68]. The global null hypothesis of no difference between groups was rejected if the test of either endpoint was statistically significant at the two-sided 0.025 level, or if both tests were significant at the two-sided 0.05 level. Differences between treatment groups were accordingly estimated with 95% confidence intervals. Exploratory subgroup analyses of the primary outcome measures were performed according to strata and sensitivity analyses were performed by exploring the effect of additional covariates thought to be of prognostic importance [69]: type of injury (aetiology), age at injury (years), time since injury (years), and reported fatigue at intervention. Secondary outcomes of self and teacher reports (raw scores BRIEF_BRI_ and BRIEF_MI_) were also analysed by the linear mixed model. Secondary outcomes pertaining performance-based neuropsychological tests at 6-month follow-up were analysed by linear regression, with baseline raw scores, treatment group, and strata as covariates. For secondary endpoints, the number of outcomes were theory-driven, pre-planned and limited (only one outcome per function) to avoid an inflation of type I error. Estimates of treatment differences were presented with 95% confidence intervals, and tests were performed using a two-sided significance level of 0.05. No adjustment of multiplicity was planned. Distributional assumptions were checked by visual inspection of residual plots. No imputation of missing scores was made. Main analysis was conducted March 2020 using SAS v9.4. IBM-SPSS version 26 and Stata16 were used for secondary analyses.

## Results

Flow chart on recruitment is presented in Fig. 1. Of the 99 individuals who were screened by interview, two declined to participate and ten did not meet the eligibility criteria: nine reported insufficient EF complaints and one was excluded for not meeting inclusion criteria for functional level. Thus, 87 were randomized. Since the interventions were group-based (assembled by age, sites, and treatment allocation), some participants experienced a latency from randomization pending initiation of a group. Nine potential participants withdrew from participation before baseline assessment while waiting start of assigned intervention group due to unforeseen changes in life situation (worsening of illness, medication testing, intensifying of physical therapy). Two were excluded post-randomization (before baseline) after identification of violations of eligibility criteria not previously communicated. Pre-inclusion attrition was evenly distributed between the two groups, 6 in pGMT and 5 in pBHW. Demographic, injury characteristics, and cognitive scores at baseline for the 76 participants are summarized in Table 1.

### Treatment compliance

Seventy-three participants (96%) completed the allocated intervention (Fig. 1). There was no attrition post-intervention, and only two participants (one in each group) failed to attend at the 6-month follow-up due to cancer recurrence, resulting in 93% attendance.

### Primary outcomes

**Fig. 3:**
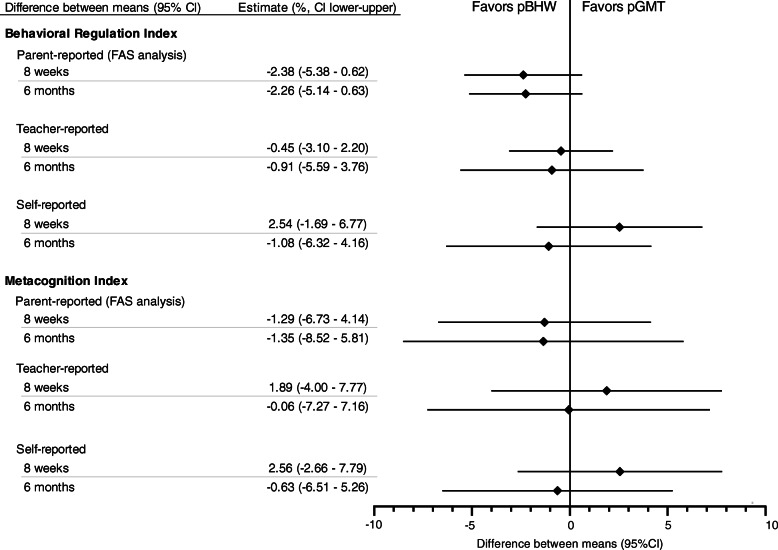
Estimated differences between treatment groups on the BRIEF questionnaire with 95% confidence intervals. BRIEF, Behavior Rating Inventory of Executive Function; pGMT, paediatric Goal Management Training; pBHW, paediatric Brain Health Workshop; FAS, full analysis set. Estimated differences (pGMT-pBHW) in mean raw scores, adjusted for baseline scores and strata; age at intervention (10 to 13 years/14 to 17 years); and study site (Trondheim/Oslo)

The results show no significant difference between the two intervention groups for the primary outcome (changes in parent-reported BRIEF raw scores from baseline (*T*_1_) to 6 months follow-up (*T*_3_)). Thus, estimated between-group differences in the linear mixed model for BRIEF_BRI_ was − 2.3 (95% CI − 5.1 to 0.6), *p* = .12, and the corresponding result for BRIEF_MI_ was − 1.4 (95% CI − 8.5 to 5.8), *p* = .71 (Fig. 3). Exploratory stratified subgroup analyses and sensitivity analyses did not differ meaningfully from the main result [see Additional file 2, 3]. Although no difference in effect for the two interventions was demonstrated, the results show significant decrease in parent-reported executive dysfunction for both interventions. In linear mixed models, the estimated changes from baseline (*T*_1_) to 6-month follow-up (*T*_3_) with 95% CIs and p values for BRIEF_BRI_ were − 3.8 (− 5.6, − 1.9, *p* < .001) for pGMT and − 5.7 (− 8.5, − 2.9, *p* < .01) for pBHW. The corresponding results for BRIEF_MI_ were − 6.9 (− 10.9, − 2.9, *p* = .001) for pGMT and − 8.9 (− 13.2, − 3.9, *p* = .001) for pBHW.

### Secondary outcomes

As presented in Fig. 3, comparing pGMT and pBHW at *T*_3_ mixed modelling revealed no difference in either self- or teacher reports for BRIEF_BRI_ or BRIEF_MI_, which supports the primary results of no difference between interventions.

Results from performance-based tests and the complex naturalistic task are presented in Table 2 and demonstrates that at 6-month follow-up, the pGMT group had improved inhibition scores (difference in *T*-score at *T*_3_, 5.2 (95% CI 1.6 to 8.7); *p* = .005) and executive attention (difference in raw score at *T*_3_, 9.9 (95% CI 2.2 to 17.6); *p* = .01), but not updating (difference in raw score at *T*_3_, − 0.2 (95% CI − 1.7 to 1.4); *p* = .83) or shifting (difference in raw score at *T*_3_, − 4.5 (95% CI − 18.9 to 9.9), *p* = .54). The pBHW enhanced performance on the complex naturalistic task (CCT) (difference in raw score at *T*_3_, − 4.3 (95% CI − 8.4 to − 0.3), *p* = .04).

**Table 2:** Estimated difference between pGMT and pBHW in performance-based tests at 6-month follow-up with 95% confidence interval

EF outcomes	pGMT, mean (SD)^a^	pBHW, mean (SD)^a^	Estimated mean difference (95% CI)^b^	*P* value
Updating, raw scores (*n* = 70)	26.2 (5.5)	25.3 (4.4)	− 0.2 (− 1.7 to 1.4)	.830
Shifting, raw scores (*n* = 70)	94.3 (57.6)	96.4 (46.2)	− 4.5 (− 18.9 to 9.9)	.540
Inhibition, *T*-scores (*n* = 71)	48.3 (9.3)	54.8 (9.6)	5.2 (1.6 to 8.7)	.005
Executive attention, raw scores (*n* = 71)	72.4 (31.7)	75.1 (22)	9.9 (2.2 to 17.6)	.010
Complex naturalistic task, raw scores (*n* = 68)	18.7 (16.4)	15.8 (13.1)	− 4.3 (− 8.4 to − 0.3)	.040

*pGMT* paediatric Goal Management Training, *pBHW* paediatric Brain Health Workshop

^a ^Unadjusted mean (SD)

^b ^Estimated mean differences from multiple linear regressions including the baseline measure as a covariate. Mean difference in raw scores except for inhibition (*T*-score), adjusted for baseline scores and strata; age at intervention (10 to 13 years/14 to 17 years) and study site (Trondheim/Oslo). Updating: Digit span, Wechsler Intelligence Scale for Children-Fifth edition (WISC-V) (raw score); shifting: Trail Making Test 4 (TMT 4, from the Delis–Kaplan Executive Function System, D-KEFS) (raw score, total time); inhibition: commissions from Conners’ Continuous Performance Test, 3ed (CPT-III) (T-scores, normative: *M* = 50, SD = 10); executive attention: Color Word Interference Test 4 (CWIT4, D-KEFS) (raw score, total time); and complex naturalistic task: Children’s Cooking Task (CCT) (raw score, total errors)

### Adverse events

One psychological reaction was reported in the pGMT group at the end of intervention. It was assessed and handled according to study protocol and good clinical practice [70]. Although symptoms originated before study inclusion, it was recorded as an adverse event (moderate) with uncertain connection to the intervention.

## Discussion

The aim of the present study was to examine the efficacy of pGMT, adapted from a metacognitive protocol proven effective in adults, for children in the chronic phase of pABI with EF complaints, when compared to psychoeducation (pBHW). Keeping non-specific factors constant, the metacognitive intervention did not demonstrate an additional effect in reducing daily life executive dysfunction. Secondary, performance-based tests, however, showed more improvement in pGMT pertaining inhibition and executive attention, while pBHW demonstrated better performance in the practical complex naturalistic task at 6-month follow-up.

Several factors may have contributed to pGMT not replicating improved daily-life EF as seen in the adult version. First, we have to consider the impact of developmental factors. The young brain has the capacity for more efficient neural restitution, by neural regrowth and anatomical reorganization [71, 72] which may give expectations of a particularly trainable period during adolescence. However, the young brain is also more vulnerable to more severe, diffuse, and enduring deficits after ABI compared to the adult brain [5, 73]. Additionally, the protracted development of EFs and metacognition may have affected metacognitive training effects [74].

When tailoring metacognitive treatments, duration of training is an important factor [19, 75] for implementation and automatization of metacognitive strategies [74]. Unlike pBHW, pGMT requires repeated practice of specific techniques. In a meta-analysis, more GMT hours (i.e., higher dose) was associated with greater positive effects on EF [19]. In the present study the participants received 14 h of hospital training, which might not have been enough time to practice and consequently consolidate and automatize the pGMT strategies to take full advantage of the method in daily life.

Age at the time of training may influence the effects of metacognitive training [74] since previous research has documented greater improvements in late adolescent and adulthood, compared to early adolescence [76, 77]. Although our study was not powered to discover age related differences, the exploratory results did not indicate any difference in effect by age at intervention. Importantly, the referred to studies demonstrating greater improvements in late adolescent included participants in a more acute rehabilitation phase (0–6 months post-insult) than the present study, as well as a more homogeneous sample consisting of only participants with TBI. Larger studies are therefore required to be able to clarify potential differences in the efficacy of pGMT in younger and older adolescents.

Being a metacognitive intervention, pGMT requires self-awareness and reflective skills. Since executive dysfunctions in pABI are associated with lower self-awareness [78], it is likely that for some participants, the high metacognitive demands in pGMT might have surpassed the available resources, thus impeded full comprehension or utilization of the strategies as seen in adults. Even though updating, inhibitory control and the ability to sustain and shift attention are approaching adult capacity in older healthy adolescents, their self-monitoring and reflective abilities may not fully mature until early adulthood [79].

Further, keeping with recommendations of contextual training and involvement of the adults in the child’s life [30, 33], which may have resulted in a more robust BHW-comparator than in the adult studies. Powerful non-specific mechanisms common to pGMT and pBHW (e.g., contact with professionals, meeting peers with similar challenges) may have resulted in improvements following both interventions. Indeed, sharing experiences and knowledge replenishment may have enhanced coping strategies or self-efficacy [80]. Further, it has been shown that individual’s expectations of efficacy moderate intervention effects [81]. This may, at least partially, explain the improvements in the active control intervention although it was not specifically aimed at EF.

Lastly, assessment of EF is a known challenge [7, 35–38] and factors related to appropriate EF assessment may have contributed to the small and non-significant differences between interventions. The age-corrected BRIEF scores at baseline were in the normal range [47], which is not in accordance with the executive difficulties described by the parents (and participants) in the screening interview used for inclusion in our study. Rating scales have been criticized for undesirable variability [82] and lack of useful information to assist the best option [83]. On the other hand, the screening interview was guided by a research nurse and the respondents were able to more freely describe the real-life EF difficulties compared to the standardized questionnaire. This notion is supported by findings in another RCT (*n* = 29) evaluating the efficacy of an intervention programme based on social mediation, cooperative learning, and metacognition in pABI [32]. Here, no improvements on the BRIEF were observed when comparing interventions, despite improved metacognitive strategies and improved self-concept assessed with other measures [32], thus questioning whether the BRIEF is sensitive enough to measure change in metacognitive function. Additionally, as parents also received the intervention, it is possible that they were made more aware of EF dysfunction in their child post-intervention in comparison to pre-intervention, potentially reporting less dysfunction at baseline and consequently biasing the estimated effect towards a lesser decrease in dysfunction at follow-up [32].

Investigating secondary intervention effects at the impairment level (performance-based tests), however, pGMT had improved inhibition and executive attention when compared to pBHW. The strong emphasis in pGMT on inhibitory control training (i.e., “stop-and-think”) may be related to the enhanced capacity for response inhibition observed in pGMT at 6-month follow-up (reduced inhibition errors), as pBHW increased in errors in the same period. These results are consistent with GMT findings in adult studies [24], suggesting enhanced bottom-up processing [23]. Further, pGMT displayed improvements in executive attention at both post-intervention assessments (when compared to pBHW), which may support underlying alterations in brain networks linked to attentional control [29]. This finding requires neuroimaging studies to be confirmed. As higher-order EF build on core processes [10], enabled by executive attention [44, 84], these enhancements could potentially improve higher-order EFs (e.g., problem solving). The improvements in inhibition and executive attention may indicate that participants in the pGMT group have initiated a response to the metacognitive intervention, although not fully completed. Future research should investigate the sequencing of potential change in EFs following metacognitive interventions, to test the hypothesis of more sensitive EFs (i.e., inhibition) affected earlier in the course compared to others requiring a more prolonged treatment. The finding of pGMT not improving updating (i.e., digit span) and shifting (i.e., TMT4) more when compared to pBHW are in accordance with findings from adult studies [50].

Despite potential enhancements in the underlying core EF processes, pGMT did not result in improved performance when compared to pBHW in the complex naturalistic task (CCT). Potential insufficient practice of pGMT strategies and/or developmental factors affecting the capacity of taking full advantage of pGMT may also have affected performance in the CCT. On the other hand, a greater reduction in errors on the CCT after pBHW suggests that psychoeducation delivered to both patients and families is beneficial [85]. Thus, enhanced knowledge of brain injury and (dys)function following pBHW may have enhanced coping strategies and self-efficacy [86].

The ecological validity of performance-based tests has been questioned [79] and consequently accelerated the development of questionnaires such as BRIEF to assess real-life behaviours [79]. However, several factors (such as under/over-reporting, social desirability bias) may also affect the accuracy and validity of questionnaires, as they involve multiple executive and non-executive processes, in addition to contextual influences (e.g., motivation), thus posing major interpretational problems and perhaps not, reflecting EF per se [87]. Questionnaires and performance-based tests correlate relatively poorly [35] and may index different underlying constructs, namely, the efficiency of cognitive abilities and success in goal pursuit [35]. Subsequently, novel ecological methods mimicking everyday impairments are warranted. CCT is a novel multitasking method that, although properties have not yet been fully examined, has demonstrated sensitivity to executive dysfunction in children with TBI [63] and, thus, represented EF assessment in a naturalistic setting. Finally, since executive dysfunction is considered to be a trans-diagnostic symptom [88], we were interested in investigating potential effects across mixed aetiologies. The significance of including a heterogeneous sample is not fully known; nevertheless, we believe it is a potential strength to the study that we tested the intervention in a heterogeneous group initially. Adherence was almost total in both interventions, even though adherence is known to be confounded by fatigue and lack of concentration [78], suggesting that both interventions were perceived as feasible and meaningful.

The study’s main limitation is the absence of a wait-list control to account for natural change over time. Previous studies have compared GMT to an active control, but comparing pGMT to pBHW may have masked potential effects of pGMT. The design of keeping non-specific factors similar in the two interventions (i.e., involvement of parents and teachers, professional attention, group dynamics) may have produced a more robust active control intervention than employed in the adult studies. Although difficult to assess, pre- and post-interventional measures of self-awareness would have been useful to clarify its possible impact on effect. Despite being larger than other paediatric studies in sample size, it is likely that the present study was underpowered to detect small differences, especially considering the comprehensive psychoeducation condition employed. The power calculations were performed using the GEC from the BRIEF, rather than the two co-primary endpoints BRI and MI, and were based on a significance level of 0.05 rather than significance levels adjusted for two co-primary outcomes. The sample size was likely to have been too small to detect small differences, if they exist, between the two treatments even if they were potentially clinically relevant. Hence, the results should be regarded as exploratory, and there is a need of future studies with larger sample sizes to confirm our findings. The present study did not explore the potential impact of injury severity on intervention effect. Since injury severity is a well-known predictor of poorer outcome in TBI research [3, 89] and previously has demonstrated impact on interventional effect [90], this should be investigated in future studies. In addition, it is important to consider that the pABI sample in our study was selected based on EF complaints. Thus, our findings may not be generalized to all survivors of pABI.

Future studies should explore subgroup differences in responses to pGMT relating to factors such as age at intervention and severity of insult. Future research should also include objective functional measures (e.g., school performance) and combinations of assessment levels to further clarify the indexed constructs. Finally, future research may consider examining treatment dose (e.g., number of sessions and total treatment time) and the potential effects of “booster sessions” [91].

## Conclusion

To our knowledge, this is one of few high-quality RCTs with blinded assessments on cognitive rehabilitation in the pABI population, addressing several methodological shortcomings from previous studies. Our main findings demonstrated that the metacognitive intervention did not yield additional effectiveness in reducing daily life executive dysfunction compared to pBHW. Moreover, both treatments were associated with distinct effects at different assessment levels of EF. This emphasizes the importance of measuring the various aspects of EF in clinical trials. Considering the high burden of executive dysfunction after pABI, the results point to encouraging clinical implications; both interventions are brief and had high rates of attendance and adherence, indicating high patient acceptance. It is recommended for future clinical trials to explore the sequences of change in EFs, as our secondary findings indicate improvements in inhibition and executive attention after pGMT, suggesting an initiation of response to the metacognitive intervention. Future research will also need to determine possible differences in effects related to treatment duration, developmental factors, and injury characteristics (e.g., severity and aetiology).

## Supplementary Information

**Additional file 1:.** Semi-structured interview.

**Additional file 2:.** Stratified analyses Treatment group difference with 95% confidence intervals.

**Additional file 3:.** Sensitivity analyses: Treatment group difference with 95% confidence intervals (Full analysis set/Parent reported).

**Additional file 4:.** Consort checklist

## Data Availability

The dataset analysed during the current study are available from the corresponding author on reasonable request.

## Abbreviations

BRI: Behavioral Regulation Index
BRIEF: Behavior Rating Inventory of Executive Function
CCT: Children’s Cooking Task
CPT-III: Conners’ Continuous Performance Test, 3ed
CWIT: Color Word Interference Test
D-KEFS: Delis–Kaplan Executive Function System
MI: Metacognition Index
pABI: Paediatric acquired brain injury
pBHW: Paediatric Brain Health Workshop
pGMT: Paediatric Goal Management Training
TBI: Traumatic brain injury
TMT: Trail Making Test
WISC-V: Wechsler Intelligence Scale for Children-Fifth edition
