# Supplementary material for: Rehabilitation of executive function in chronic paediatric brain injury: a randomized controlled trial
Source: BMC Med. 2021 Nov 2;19:253. doi: 10.1186/s12916-021-02129-8 (PMC8561897; doi:10.1186/s12916-021-02129-8)
Supplement: Supplementary file 1 — Additional file 1:. Semi-structured interview. [file 12916_2021_2129_MOESM1_ESM.pdf]

Additional file 1: Description of the screening process and the semi-structured interview
Screening of participants was conducted in four steps

1) Identification: Potential participants were identified based on discharge diagnosis and hospital record information. The following ICD-10 codes (WHO) were utilized: B00.3, B00.4, B02.0, B02.1, A85, A86, G04, G05, F07.1 (Inflammation); C71, C72 (Brain tumor); G93.1/R09.0, T75.1, T90.1 (Anoxia/hypoxia); G93.6, I60- I69 (Stroke); S02, S06-S09, T90 (TBI); F07.2, T90 (Post-commotio-syndrome). In addition to discharge diagnosis, participants were identified on the basis of inclusion in the standardized patient care for children with brain tumours and for children with acquired brain injury implemented at the two research sites. At identification, each individual journal was inspected using a pre-set data extraction sheet to systematize and ensure proper injury information and diagnosis across sites.

2) **Invitation**: Following a positive identification, potential participants received a written invitation.

3) **Brief first telephone contact**: Recipients of an invitation contacted the project or a research nurse familiar with the patient group, contacted those who had not initiated contact. During this first contact, 56 declined to participate (e.g. not motivated, too time consuming or tiring) or no reasons were given. Further, 46 were considered not eligible (36 reported not having executive difficulties, 7 reported functional levels not compatible with participation, thus meeting exclusion criteria and 3 reported other reasons (e.g. other health complications preventing participation). For those considered eligible for the screening interview and who expressed interest in participation, an individual telephone-appointment was made.

4) **Screening interview**, a semi-structured interview with a pre-set interview manual including the following themes:

- demographics
- clarification of language skills (ability to follow group training)
- school (incl. grade) and clarification of any special needs in school
- aetiology/injury and treatment
- allergies (due to the cooking task)
- functional levels (motor, vision, hearing, fatigue, reading, writing)
- medical conditions (e.g. neurological disorders, degenerative diseases, seizures)
- developmental disorders (e.g. Tourettes, ASD, ADHD)
- psychiatric diagnoses (e.g. anxiety, depression, receiving treatment)
- medication
- a free description of the child/adolescent`s function in daily-life (home, school) in relation to goal attainments (tasks/chores), attentional capacities, and regulation
- specific evaluation of executive function by questions compiled by experienced neuropsychologists to guide assessment (current function vs pre-injury function) (e.g. "*Does the child handle doing more than one thing at the same time? Does the child manage to plan activities? Does the child manage to motivate himself/herself? How does the child regulate emotions? Is the child easily distracted?*)
- Clarification of ability to attend group training

The interview was conducted with primary caregiver or participants surpassing 16 years. In reports of executive difficulties, the interviewer always asked for specific examples. If in doubt, an at-site neuropsychologist was consulted.

5) **Inclusion**: Successively, individuals considered eligible and with a provided informed written consent, were designated a study number and randomly assigned by a computer-based algorithm in a Web Clinical Research Form (webCRF) was set up for randomization by an independent allocator.
