# Supplementary figures and images for: Rehabilitation of executive function in chronic paediatric brain injury: a randomized controlled trial
Source: BMC Med. 2021 Nov 2;19:253. doi: 10.1186/s12916-021-02129-8 (PMC8561897; doi:10.1186/s12916-021-02129-8)

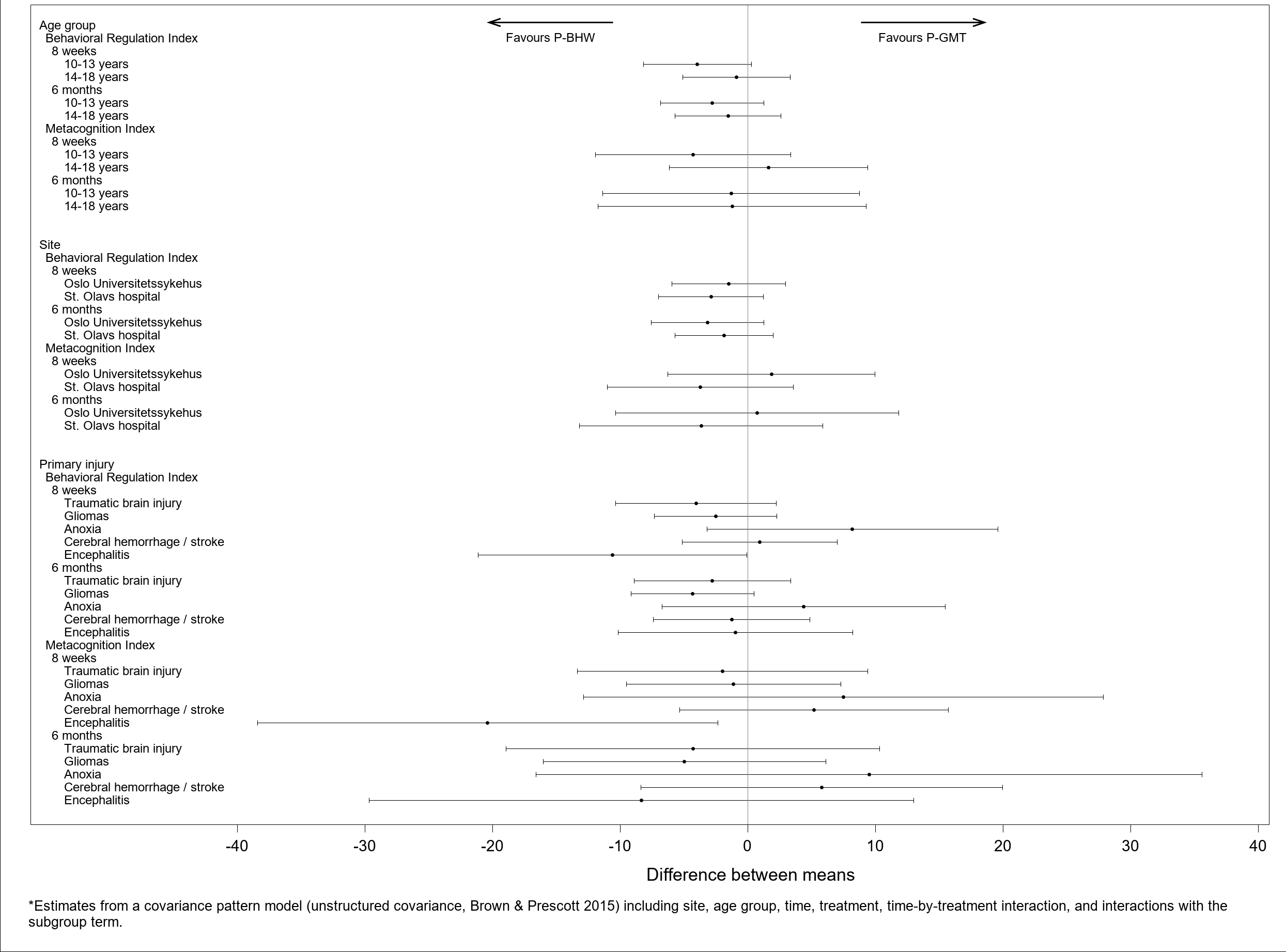

Supplement: Supplementary file 2 — Additional file 2:. Stratified analyses Treatment group difference with 95% confidence intervals. [file 12916_2021_2129_MOESM2_ESM.tif]

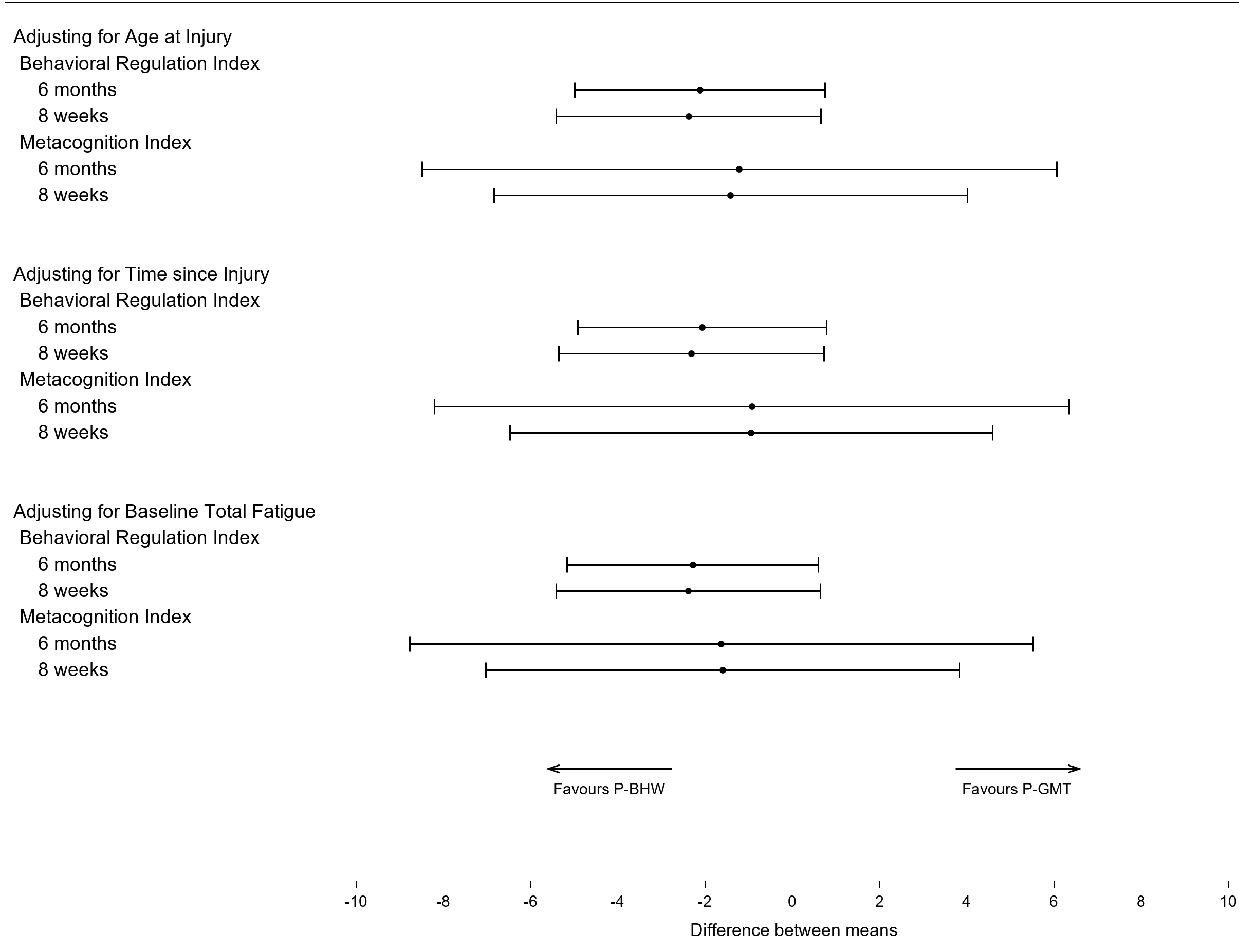

Supplement: Supplementary file 3 — Additional file 3:. Sensitivity analyses: Treatment group difference with 95% confidence intervals (Full analysis set/Parent reported). [file 12916_2021_2129_MOESM3_ESM.tif]
